# NeuRank: learning to rank with neural networks for drug–target interaction prediction

**DOI:** 10.1186/s12859-021-04476-y

**Published:** 2021-11-26

**Authors:** Xiujin Wu, Wenhua Zeng, Fan Lin, Xiuze Zhou

**Affiliations:** 1 School of Informatics, Xiamen University, Xiamen, China; 2 Shuye Technology Co., Ltd., Hangzhou, China

**Keywords:** Drug–target interactions, Drug discovery, Neural network, Ranking task

## Abstract

**Background:**

Experimental verification of a drug discovery process is expensive and time-consuming. Therefore, recently, the demand to more efficiently and effectively identify drug–target interactions (DTIs) has intensified.

**Results:**

We treat the prediction of DTIs as a ranking problem and propose a neural network architecture, NeuRank, to address it. Also, we assume that similar drug compounds are likely to interact with similar target proteins. Thus, in our model, we add drug and target similarities, which are very effective at improving the prediction of DTIs. Then, we develop NeuRank from a point-wise to a pair-wise, and further to list-wise model.

**Conclusion:**

Finally, results from extensive experiments on five public data sets (DrugBank, Enzymes, Ion Channels, G-Protein-Coupled Receptors, and Nuclear Receptors) show that, in identifying DTIs, our models achieve better performance than other state-of-the-art methods.

## Introduction

In drug discovery, experimental verification of Drug–Target Interactions (DTIs) is so expensive and time-consuming that only a small fraction of DTIs have been verified [1–6]. Therefore, there is a great need for an effective and efficient computational method for identifying DTIs.

Recently, with the rapid development of high-throughput techniques, a great deal of drug–target interaction data has been generated [7, 8]. Traditional experimental verification limits the speed at which new drugs can be identified [9–11]. To meet the increasing need for rapid and effective drug discovery, machine learning methods have become more and more widely applied to detect potential DTIs from verified DTI information [12–16]. Matrix Factorization (MF) [17], one of the most successful methods in recommender systems [18], has been widely extended to DTI prediction. For example, Cobanoglu et al. [19] adopted Probabilistic Matrix Factorization (PMF) [20] to identify the potential drug–target association between chemicals and targets; Gönen [21] developed MF by adopting chemical and genomic kernels to predict DTI networks; Liu et al. [9] added neighborhood regularization to logistic MF to predict the probability that a drug will interact with a target. However, most existing MF-based methods only considered a linear and shallow relation between a drug and a target, which is insufficient to capture the complicated relationship between them.

Recently, great success has been achieved with deep learning models in Computer Vision (CV) [22, 23], Neural Language Processing (NLP) [24, 25], and recommender systems [26–28]. The goal of deep learning models is to capture the higher-order relation between input data by their hidden layers [3, 29, 30]. To overcome the limitation of traditional MF-based methods, many researchers have tried to apply deep learning models to the prediction of DTIs. For example, Wang et al. [31] adopted Restricted Boltzmann Machines (RBM) [32] to predict DTIs; Gao et al. [33] proposed a neural network combined with a two-way attention network to provide biological insights to interpret the drug–target predictions; Altae-Tran et al. [34] integrated Long Short-Term Memory (LSTM) and graph Convolutional Neural Networks (CNN) to obtain meaningful information from a few data points. Compared with MF, deep learning models have a greater ability to capture deep representation from raw input data.

Although many deep learning models have been proposed to predict potential DTIs, little effort has been devoted to explore ranking learning in the prediction of DTIs. To comply with the DTI prediction setting, Peska et al. [35] extended Bayesian Personalized Ranking (BPR) [36], which has shown excellent performance in various learning tasks; Yuan et al. [37] designed a ranking-based ensemble learning method, DrugE–Rank, which is modeled on multiple well-known similarity-based methods to improve prediction performance. But, these methods, based on traditional machine learning methods, such as MF and k-Nearest Neighbor (kNN), are insufficient to capture the drug–target latent structures, for they do not consider any deep interactions between latent features.

Inspired by the good performance of deep learning models in various tasks, to predict DTIs, we designed a neural network architecture, NeuRank, in which, we treat identifying DTIs as a ranking task. Deep learning models are powerful and flexible for learning useful representations. Based on Multilayer Perceptron (MLP) architecture, we extended a new interaction module for drugs and targets to better model their relationship. Then, for better performance, we developed our model from a point-wise to a pair-wise and further to a list-wise method. In the pair-wise method, we assume that the observed DTIs, which have been experimentally verified, are more trustworthy and more important than the unknown ones. Thus, we model the relative ordering from each pair of targets to make predictions, and learn to rank by optimizing a pair-wise loss function to find the correct ranking for all targets. And in the list-wise method, we seek to maximize the top-one probability of targets in the ranking list.

Many works have shown that drugs with similar chemical structures have similar therapeutic functions [38–40]. This information is used to enrich latent factors and strengthen the presentation ability of the models. For example, Zheng et al. [38] proposed a model, Multiple Similarities Collaborative Matrix Factorization (MSCMF), which learns low-rank features first and then combines them with weighted similarity matrices over drugs and targets for prediction; Zhang et al. [41] adopted drug feature-based and disease semantic similarities as constraints for drugs and diseases; Laarhoven et al. [42] using chemical similarity and interaction information about known compounds, applied the nearest neighbor algorithm to construct an interaction score for drugs. The methods with similar information are able to make better predictions than other methods without any additional information. Thus, for better build relationships between drug–drug and target–target, a similarity calculation method is used to learn the link between these data.

Our contributions are summarized as follows:

1. We solved the DTI problem by using neural networks with a strong ability to capture non-linearity from raw data and learn deep features from a ranking learning perspective;
2. To better predict DTIs, especially for new drugs and targets, we added drug–drug and target–target similarities to our model;
3. For different applications, we developed three neural networks from point-wise to pair-wise learning and further to list-wise learning.

The rest of the paper is organized as follows: “Related work” section briefly reviews the background and some related work. “Proposed methods” section presents our proposed models in detail. “Experiments” section describes the experimental results for several data sets to show the performance of our models. “Conclusion” section gives the conclusion and provides future directions.

## Related work

First, we discuss the problem to be solved and define the notations that are used in the rest of the paper. Then, we introduce two MF-based methods, which are closely related to our model: a traditional one Collaborative Matrix Factorization (CMF), and a pair-wise ranking learning one, BPR.

### Problem definition

Given a DTI matrix, $\varvec{Y} \in {\mathbb {R}}^{n\times m}$, with a set of *n* drugs, $\varvec{D}$, and a set of *m* targets, $\varvec{T}$, and element, $y_{dt} \in \left\{ 0,1 \right\}$. If drug, *d*, has been experimental verified to interact with target, *t*, then $y_{dt}=1$; otherwise, $y_{dt}=0$. $\varvec{P}\in {\mathbb {R}}^{n\times k}$ and $\varvec{Q} \in {\mathbb {R}}^{m\times k}$ denote the low-rank latent features of drugs and targets, respectively, where *k* denotes the number of latent features. $\varvec{p}_d$ and $\varvec{q}_t$ denote the latent features of drug, *d*, and target, *t*, respectively. The goal of MF for DTIs is to learn $\varvec{P}$ and $\varvec{Q}$ to reconstruct $\varvec{Y}$:

1 $$\begin{aligned} arg \min \limits _ { \varvec{p},\varvec{q}} \sum _{(d,t) \in \varvec{V}} \left( y_{dt}-\varvec{p}_{d}\varvec{q}_{t}^T \right) ^2 + \lambda \left( \left\| \varvec{P} \right\| ^2_{F} + \left\| \varvec{Q} \right\| ^2_{F} \right) \end{aligned}$$

where $\varvec{V}$ denotes the set of interactions that have been experimentally verified; $\left\| \cdot \right\| ^2_F$ denotes the Frobenius norm; $\lambda$ denotes a regularization coefficient.

### CMF

CMF, proposed in [38], adopts multiple kinds of drug–drug and target–target similarities. The objective function of CMF is defined as follows:

2 $$\begin{aligned} \begin{aligned} arg \min \limits _ { \varvec{p},\varvec{q}}&\sum _{(d,t) \in \varvec{V}} \left( y_{dt}-\varvec{p}_{d}\varvec{q}_{t}^T \right) ^2 + \lambda \left( \left\| \varvec{P} \right\| ^2_{F} + \left\| \varvec{Q} \right\| ^2_{F} \right) \\&+ \lambda _{d} \left\| \varvec{S}^d - \varvec{PP}^T\right\| ^2_{F} + \lambda _{t} \left\| \varvec{S}^t - \varvec{QQ}^T\right\| ^2_{F} \end{aligned} \end{aligned}$$

where $\lambda$, $\lambda _d$, and $\lambda _t$ denote regularization coefficients; $\varvec{S}^d \in {\mathbb {R}}^{n\times n}$ denotes the similarity matrix for drugs, and $\varvec{S}^t \in {\mathbb {R}}^{m\times m}$ denotes the similarity matrix for targets.

The first term, MF, learns low-rank latent features, $\varvec{P}$, and, $\varvec{Q}$, to reconstruct $\varvec{Y}$; the second term is L2 regularization to prevent the model from over-fitting; the last two terms are regularizations, which minimize the squared error between $\varvec{S}^d$ and $\varvec{PP}^T$, and between $\varvec{S}^t$ and $\varvec{QQ}^T$. The key idea is that the similarity between drugs or targets should be approximated by the inner product of the corresponding two feature vectors.

### BPR

DTIs provide only very few verified instances to train; therefore, it is inherently difficult to uncover the interaction probability between drugs and targets. Instead of directly predicting the absolute probability of DTIs, BPR uses pair-wise ranking loss to model the relative order between observed and unobserved interactions.

Based on BPR, Peska et al. [35] developed the DTI prediction model, which has shown promising power in personalized recommendations. The key idea of BPR is that observed interactions should be ranked higher than unobserved ones [36]. The goal of BPR for DTI predictions is to learn the probability that a drug will interact with a target. BPR aims to maximize the posterior probability that drug, *d*, interacts with the pair targets of *t* and *i*: $p\left( \varvec{\theta } | t>_d i \right)$, where $\varvec{\theta }$ is the set of learning parameters. The posterior probability is defined as follows:

$$\begin{aligned} p\left( \varvec{\theta } | t>_d i \right) \propto p\left( t>_d i \right| \varvec{\theta }) \cdot p\left( \varvec{\theta } \right) \end{aligned}$$

Then, the probability that drug, *d*, interacts with target, *t*, rather than *i* is defined as follows:

3 $$\begin{aligned} \begin{aligned} p\left( t>_d i \right| \varvec{\theta })&=\sigma \left( {\widehat{y}}_{dti} \right) ,\\ {\widehat{y}}_{dti}&={\widehat{y}}_{dt}-{\widehat{y}}_{di}. \end{aligned} \end{aligned}$$

where $\sigma (x)=1/\left( 1+exp(-x)\right)$ is the sigmoid function, and ${\widehat{y}}_{dt}$ and ${\widehat{y}}_{di}$ are the predicted scores for targets *t* and *i* with drug, *d*, respectively. ${\widehat{y}}_{dt}$, estimated by MF, linearly combines drug and target features as follows:

4 $$\begin{aligned} {\widehat{y}}_{dt}=\varvec{p}_{d}\varvec{q}_{t}^T \end{aligned}$$

where $\varvec{p}_{d}$ and $\varvec{q}_{t}$ denote the latent features of drug, *d*, and target, *t*, respectively.

Finally, based on Bayesian inference, the objective function of BPR, which minimizes the pair-wise ranking loss for all pair instances, is defined as follows:

5 $$\begin{aligned} {\mathcal {L}}=-\sum _{\left( d,t,i\right) \in \varvec{F} }ln\sigma \left( {\widehat{y}}_{dt}-{\widehat{y}}_{di}\right) + \lambda \Vert \varvec{\theta } \Vert ^2_F \end{aligned}$$

where $\varvec{F}=\left\{ (d,t,i) |d \in \varvec{D} \wedge t \in \varvec{V}_d^+ \wedge i \in \varvec{V}_d^- \right\}$ denotes that drug, *d*, tends to interact with target, *t*, rather than *i*, where, when given a drug, *d*, $\varvec{V}_d^+=\{t \in \varvec{T}|y_{dt}=1 \}$ denotes a set of targets that have been experimentally verified to interact with *d*. $\varvec{V}_d^-$ is the rest, and $\lambda$ is the regularization parameter.

Both CMF and BPR are MF-based methods, which are linear in nature. Therefore, when compared to nonlinear methods, they have limited performance [27, 43]. Inspired by the idea from BPR for ranking learning in DTI prediction and the good performance of NeuMF [43] in recommender systems, we developed a neural network to promote DTI prediction in ranking perspective.

## Proposed methods

Methods for one-class data, i.e. data with only positive examples, are classified into three categories: point-wise regression, pair-wise, and list-wise methods. Point-wise regression methods directly optimize the absolute value of binary interaction. Pair-wise ranking methods assume that drugs have a higher possibility to interact with verified targets rather than unverified ones. And list-wise ranking methods seek to maximize the top-one probability of targets in the ranking list.

In this section, we build our NeuRank to learn simultaneously the latent features of DTIs and similarity information. First, we introduce in detail the framework of the point-wise method, NeuRank. Then, we develop our model from point-wise to pair-wise learning and further to list-wise learning. The purpose of our models is to predict the probability that a drug will interact with a target from observed DTIs.

### Framework

**Fig. 1:**
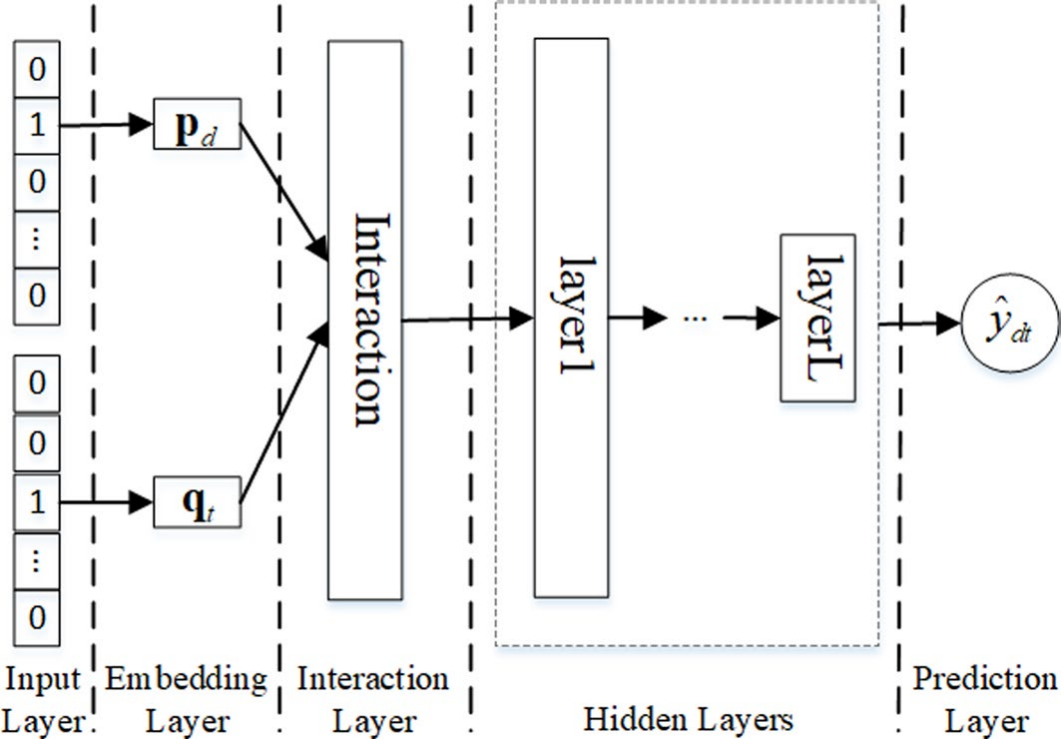
Framework of NeuRank. NeuRank, a point-wise network, consists of the five layers: input, embedding, interaction, hidden, and prediction

Point-wise methods, which consider unobserved interactions to be inherently negative, combine the latent features of drugs and targets to predict the score used to rank. Figure 1 illustrates the network framework of NeuRank, which consists of the following five layers: input, embedding, interaction, hidden, and prediction.

**Input and embedding layers** The role of the embedding layer is to transfer drug and target IDs from the input layer to latent representation space and map the sparse features to dense features as follows:

6 $$\begin{aligned} \varvec{p}_d=\varvec{P}^T \varvec{E}_d \end{aligned}$$

7 $$\begin{aligned} \varvec{q}_t=\varvec{Q}^T \varvec{E}_t \end{aligned}$$

where $\varvec{P}\in {\mathbb {R}}^{n\times k}$ and $\varvec{Q} \in {\mathbb {R}}^{m\times k}$ denote the embedding matrices for drugs and targets, respectively; *d* and *t* denote the one-hot encoding representation of the ID of a drug and a target, respectively, and their embedding vectors $\varvec{q}_d \in {\mathbb {R}}^{1\times k}$ and $\varvec{q}_t\in {\mathbb {R}}^{1\times k}$, respectively.

**Interaction layer** The role of the interaction layer is to model the interactions between drugs and targets in the shallow layer. The interaction layer, which captures the row-rank relations between drugs and targets, is defined as follows:

8 $$\begin{aligned} \varvec{h}_0=f\left( \varvec{p}_{d},\varvec{q}_{t} \right) \end{aligned}$$

where $f(\cdot )$ denotes the interaction functions between $\varvec{p}_u$ and $\varvec{q}_i$, such as concatenation, element-wise product, and element-wise sum. We chose element-wise product as our interaction function.

**Hidden layers** The role of the hidden layers is to learn nonlinear correlations between drugs and targets. Hidden layers provide neural networks a powerful ability to model the high-rank relationships between features as follows:

9 $$\begin{aligned} \begin{aligned} \varvec{h}_1&=a\left( \varvec{W}_{1}^T\varvec{h}_{0}+\varvec{b}_1 \right) \\&\cdots \\ \varvec{h}_{L}&=a\left( \varvec{W}_{L}^T\varvec{h}_{L-1}+\varvec{b}_L \right) \end{aligned} \end{aligned}$$

where $\varvec{W}_l$, $\varvec{b}_l$, $\varvec{h}_{l}$ and $a(\cdot )$ denote weight, bias, output, and activation functions of the *l*-th ($0< l\le L$) layer, respectively. The ReLU function is used as our activation function.

**Prediction layer** The role of the prediction layer is to compute the probability that a drug will interact with a target. The output, ${\widehat{y}}_{dt }$, is defined as follows:

10 $$\begin{aligned} {\widehat{y}}_{dt}=\sigma (\varvec{W}_{L+1}^T\varvec{h}_{L}+\varvec{b}_{L+1}) \end{aligned}$$

where $\sigma (\cdot )$ denotes the sigmoid function.

In NeuRank, the square loss function is used to evaluate loss and the L2 norm is used to regularize all learning parameters:

11 $$\begin{aligned} {\mathcal {L}}_1=\sum _{(d,t) \in \varvec{V}}\left( y_{dt} - {\widehat{y}}_{dt} \right) ^2 + \lambda \Omega (\varvec{\Theta }), \end{aligned}$$

where $\varvec{\Theta }$ denotes the learning parameter set of NeuRank.

### Pair-wise NeuRank

**Fig. 2:**
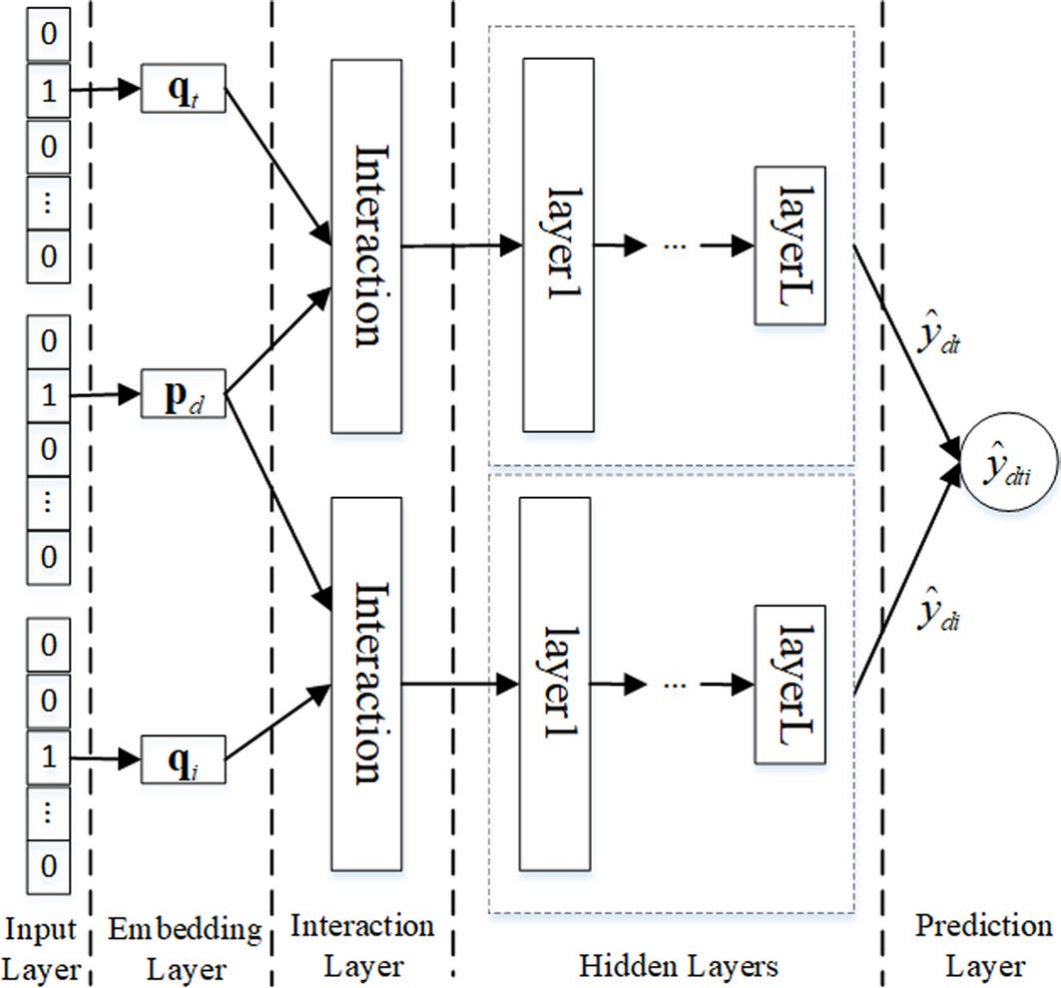
Framework of pNeuRank. pNeuRank, a pair-wise method, assumes that observed interactions are more trust worthy than unobserved ones. It consists of the five layers: input, embedding, interaction, hidden, and prediction

To make predictions, pair-wise methods model the relative ordering from each pair of targets. In contrast to the point-wise method, pair-wise methods assume that observed interactions are more trust worthy than unobserved ones. Then, NeuRank is developed from point-wise to pair-wise learning NeuRank (pNeuRank). Illustrated in Fig. 2 is the network framework of pNeuRank.

In pNeuRank, we assume that an experimentally verified target that interacts with a drug will be assigned a higher value than an unverified target. Thus, the objective function is defined as follows:

12 $$\begin{aligned} {\mathcal {L}}_2=-\sum _{\left( d,t,i\right) \in \varvec{F} }ln\sigma \left( {\widehat{y}}_{dt}-{\widehat{y}}_{di}\right) + \lambda _p \Omega \left( \varvec{\Theta }_p\right) , \end{aligned}$$

where $\varvec{F}=\left\{ (d,t,i) |d \in \varvec{D} \wedge t \in \varvec{V}_d^+ \wedge i \in \varvec{V}_d^ - \right\}$ denotes that drug, *d*, tends to interact more with target, *t*, than with *i*; $\lambda _p$, $\lambda _d$ and $\lambda _t$ are the regularization parameters; and $\varvec{\Theta }_p$ denotes the learning parameter set of pNeuRank.

In pNeuRank, the first four layers (input, embedding, interaction, and hidden) are the same as in the previous NeuRank framework. The key difference is the final output layer, ${\widehat{y}}_{dti}$, defined as follows:

13 $$\begin{aligned} {\widehat{y}}_{dti}=\sigma ({\widehat{y}}_{dt}-{\widehat{y}}_{di}) \end{aligned}$$

where ${\widehat{y}}_{dt}$ is the output of the final hidden layer when given an observed interaction between drug, *d*, and target, *t*; ${\widehat{y}}_{di}$ is the output when given an unobserved interaction between drug, *d*, and target, *i*; and $\sigma (\cdot )$ denotes the sigmoid function to bound the gap between the two values.

### List-wise NeuRank

**Fig. 3:**
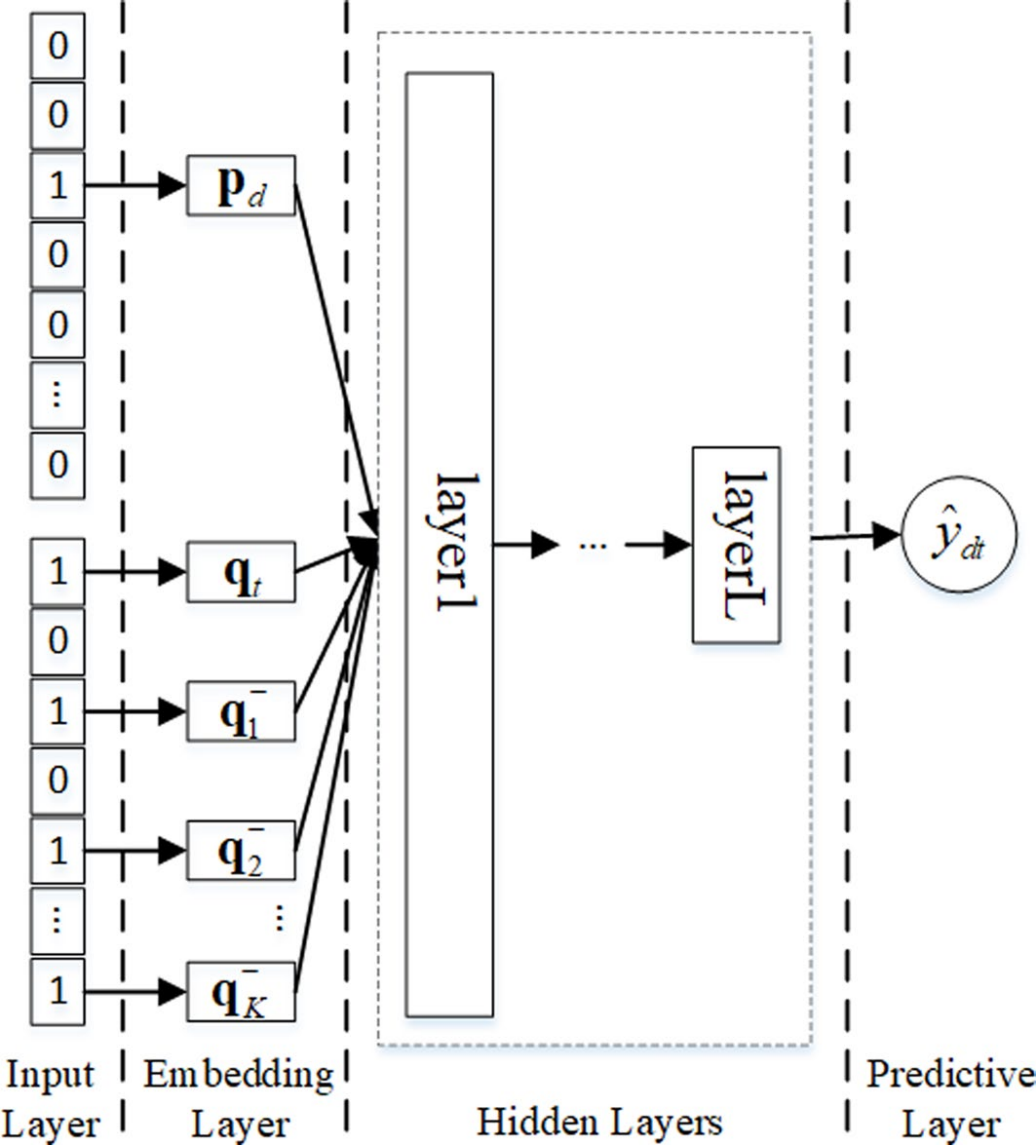
Framework of lNeuRank. lNeuRank seeks to maximize the top-one probability of targets in the ranking list. It consists of the five layers: input, embedding, interaction, hidden, and prediction

Finally, we design a list-wise framework, lNeuRank, to predict the potential DTIs. In lNeuRank, we seek to maximize the top-one probability of targets in the ranking list. The framework is shown in Fig. 3. In Fig. 3, in the list of $\left( K+1\right)$ targets for training, there are one positive instance, and *K* negative instances sampled from drug *d*. ${\varvec{q}}_i^{\_}$, where $i \in \left[ 1,K\right]$, denotes the embeddings from negative instances.

Similarly, in lNeuRank, the first four layers (input, embedding, interaction, and hidden) are the same as in the previous NeuRank framework. The key difference is the final output layer, ${\widehat{y}}_{dt}$, defined as follows:

14 $$\begin{aligned} {\hat{y}}_{dt}=softmax(x_{dt}), \end{aligned}$$

where $x_{dt}$ denotes the output from the final hidden layer. We chose the softmax function to map the results from the hidden layer to prediction. The probability ${\hat{y}}_{dt}$ that target *t* ranks at the top-one for drug *d* is defined as follows:

15 $$\begin{aligned} {\hat{y}}_{dt}=\frac{e^{x_{dt}}}{\sum _{i=1}^{K+1} e^{x_{di}}}. \end{aligned}$$

Then, loss is evaluated by cross entropy, which used to measure the distribution between the true list and the predicted list from the ranking model, is defined as follows:

16 $$\begin{aligned} {\mathcal {L}}_3=-\sum _{d=1}^n \left( \sum _{t \in l_d^+} log{\hat{y}}_{dt} + \sum _{i \in l_d^-}log\left( 1-{\hat{y}}_{di}\right) \right) + \lambda _l \Omega \left( \varvec{\Theta }_l\right) , \end{aligned}$$

where $l_d^+$ and $l_d^-$ denote the verified and unverified interaction list of drug *d*, respectively; and $\varvec{\Theta }_l$ denotes the learning parameter set of lNeuRank.

### Similarity information

Based on the assumption that similar drugs will interact with similar targets, and vice versa, we added drug–drug similarity and target–target similarity networks to our model. The chemical structure similarity between compounds and the sequence similarity between target proteins are critical for improving the prediction of DTIs, especially when few DTIs are available. Therefore, to predict the interaction from new drugs/targets, we added that similarity information to our models. Similarity regularization is defined as follows:

17 $$\begin{aligned} {\mathcal {L}}_s=\lambda _{d}\Omega ( \theta ^d) + \lambda _{t}\Omega \left( \theta ^t \right) \end{aligned}$$

where $\Omega \left( \cdot \right)$ is the function to measure the distance between predicted and true similarities. An function which measures the distance from the true values as shown in the following:

18 $$\begin{aligned} \Omega ( \theta ^d ) =\left\| \varvec{S}^d - \varvec{PP}^T\right\| ^2_{F}\end{aligned}$$

19 $$\begin{aligned} \Omega \left( \theta ^t \right) = \left\| \varvec{S}^t - \varvec{QQ}^T\right\| ^2_{F} \end{aligned}$$

Finally, the objective function is defined as follows:

20 $$\begin{aligned} {\mathcal {L}}'={\mathcal {L}}_i+{\mathcal {L}}_s \end{aligned}$$

where ${\mathcal {L}}_i$ is the loss function of NeuRank Eq. 11, pNeuRank Eq. 12, lNeuRank Eq. 16, respectively.

### Sampling for imbalance data

Since only a small fraction of DTIs is verified, which causes the imbalance data problem, i.e. the number of known DTIs is much larger than the number of unknown DTIs. The imbalance data used to train model will lead to poor performance.

To alleviate this problem, negative sampling, an effective method, is used. In general, the negative sample is proportional to the number of positive sample for each drug/target. The negative DTIs are randomly selected from a set of unobserved DTIs with an equal probability.

## Experiments

First, we introduce the data sets used in our experiments; then, we present the baselines we used as comparisons with our models and the metrics we adopted for evaluation; finally, we conduct the experiments in detail and make a detailed analysis.

### Experimental setting

**Data sets** We performed experiments on five public data sets: DrugBank, Nuclear Receptors, G-Protein-Coupled Receptors (GPCRs), Ion Channels and Enzymes. The first data set, which contains information on drugs and targets created and maintained by the University of Alberta and The Metabolomics Innovation Centre, is available at DrugBank Database^1^. As both a bioinformatics and a cheminformatics resource, DrugBank combines detailed drug (i.e. chemical, pharmacological and pharmaceutical) data with comprehensive drug target (i.e. sequence, structure, and pathway) information [44]. And the rest data sets, whose observed DTIs were extracted from public databases KEGG BRITE [45], BRENDA [46], SuperTarget [47], and DrugBank [48], are available at: http://web.kuicr.kyoto-u.ac.jp/supp/yoshi/drugtarget/. The drug chemical structure information is retrieved from the KEGG LIGAND [45], and the three-dimentional structure of target protein is retrieved from PDB [49]. Each one contains three types of information: 1) verified DTIs; 2) drug similarities; and 3) target similarities [50]. Table 1 lists some statistics about the verified DTIs in all the data sets.

**Table 1:** Statistics about data sets

Data sets	# of Drugs	# of Targets	# of Interactions
DrugBank	5018	2325	15,140
Enzymes	445	664	2926
Ion channels	210	204	1476
GPCRs	223	95	635
Nuclear receptors	54	26	90

The statistics information contains names of data sets, number of drugs, number of targets, and number of interactions

Drug–drug similarities are computed by SIMCOMP [51], which uses a graph method to model the size of the common substructures between two compounds. Target–target similarities are computed by normalized Smith-Waterman [52], which measures the similarity scores between the amino acid sequences of two proteins.

**Evaluation metrics** Following previous works [1, 9, 35, 38], two popular metrics: Area Under the Precision–Recall (AUPR) and Area Under the Curve (AUC), are used for performance evaluation in the prediction of DTIs. To evaluate our proposed methods, we used 10–fold Cross Validation (CV) and compared it with other baseline approaches. In 10–fold CV, the data set is randomly divided into 10 equal sized subsets. Of the 10 subsets, a single subset is retained as the validation data for testing the model; the remaining 9 subsets are used as training data. CV is then repeated 10 times, with each of the 10 subsets used exactly once as the validation data. The 10 results are then averaged to produce a single estimation. An AUC score is estimated in each repetition of CV; finally, the average score over all five repetitions is determined. The AUPR score is estimated in the same way.

In DTIs tasks, the main purposes are to effectively detect potential DTIs and discover new drugs. Thus, we conducted CV under the following two different settings:

$CV_{dt}$: **CV on drug–target pairs** In this case, we randomly chose 90% of the drug–target pairs in $\varvec{Y}$ as training data and the remaining 10% as testing data;

$CV_{nd}$: **CV on new drugs** In this case, we randomly chose 90% of the rows in $\varvec{Y}$ as training data and the remaining 10% as testing data;

**Baseline approaches** To illustrate the effectiveness of our models, we compared our models with the following methods:

- **PMF**, the probabilistic MF, uses dot products on the latent features of drugs and targets to make predictions [19];
- **CMF**, the state-of-the-art MF-based method, models on, not only DTIs, but also drug–drug and target–target similarities [38];
- **BRDTI**, the state-of-the-art BPR-based method, extends the BPR method by adding similarity information and target bias [35];
- **RBM**, a shallow neural network-based method for DTI prediction, its visible units encode observed types of DTIs, and its hidden units represent latent features describing DTIs [31];
- **DeepDTIs**, the state-of-the-art deep learning method, uses Deep Belief Networks (DBN) to predict DTIs, without taking similarity information into consideration [29].

**Parameter settings** Our models have seven key parameters: latent feature size (*k*), learning rate ($\tau$), the number of hidden layers (*l*), batch size (*b*), one regularization parameter for learning parameters ($\lambda$), and two regularization parameters for similarity information ($\lambda _d$ and $\lambda _t$). These parameters and factors were determined by grid-search on the validation error. In grid-search, *k* is chose from $\{8, 16, 32, 64, 128\}$; $\tau$ is chose from $\{10^{-4}, 10^{-3}, 10^{-2}, 10^{-1}\}$; *l* is chose from $\{1, 2, 3, 4, 5\}$; *b* is chose from $\{64, 128, 256, 512\}$; $\lambda$, $\lambda _d$, and $\lambda _t$ are chose from $\{10^{-4}, 10^{-3}, 10^{-2}, 10^{-1}, 1\}$. And the Adam optimizer is chose to optimize our objective function.

**Table 2:** AUC and AUPR values of all methods on five data sets under the setting $CV_{dt}$

Data sets		PMF	CMF	RBM	BRDTI	DeepDTIs	NeuRank	pNeuRank	lNeuRank
DrugBank	AUC	0.8132	0.8623	0.7778	0.8685	0.8761	0.8941	0.8983	**0.9007**
	AUPR	0.6278	0.7866	0.6984	0.8272	0.8409	0.8423	0.8456	**0.8488**
Enzymes	AUC	0.8410	0.8785	0.7833	0.8834	0.9067	0.9483	0.9502	**0.9539**
	AUPR	0.7042	0.7488	0.6693	0.7329	0.7547	0.7579	0.7602	**0.7633**
Ion Channels	AUC	0.8422	0.8974	0.7923	0.9234	0.9417	0.9661	0.9678	**0.9686**
	AUPR	0.7693	0.8034	0.7045	0.8255	0.8478	0.8389	0.8469	**0.8493**
GPCRs	AUC	0.8015	0.8244	0.7539	0.8487	0.8603	0.8561	0.8595	**0.8615**
	AUPR	0.5292	0.5435	0.4866	0.5542	**0.5778**	0.5461	0.5548	0.5532
Nuclear Receptors	AUC	0.7444	0.7637	0.6885	0.7962	**0.8043**	0.7867	0.7890	0.7832
	AUPR	0.3127	0.3463	0.2589	0.3644	0.3885	**0.4736**	0.4501	0.4378

### Results and analysis

**Overall performance** First of all, some experiments involved investigation to verify the performance of our methods on different data sets. Table 2 shows the AUC and AUPR scores obtained from all the methods under the setting $CV_{dt}$.

As shown in Table 2, in most cases, performances of all our models are higher compared with the results of other baseline approaches on the same data set. Also, lNeuRank attains the best AUC and AUPR values over the large data sets (DrugBank, Enzymes, and Ion Channels). On DrugBank, Enzymes, and Ion Channels, in terms of AUC, lNeuRank achieves 2.81%, 5.21% and 2.86% higher than the best baseline method, DeepDTIs, respectively; and in terms of AUPR, lNeuRank achieves 0.94%, 1.14% and 0.18% higher than DeepDTIs, respectively. These results indicate that, in the large data sets, when using neural networks, our model makes high quality predictions.

From the results shown in Table 2, we conclude the following: (1) on the large data sets, lNeuRank >pNeuRank >NeuRank, which indicates that large data sets contain sufficient ranking information for our models to learn accurate features; (2) on the two smallest data sets (GPCRs and Nuclear Receptors), our models achieve worse results than DeepDTIs for these two cases, and a common trend in all cases is NeuRank >pNeuRank >lNeuRank. The best possible reason is that both data sets are too small to contain enough information to make a ranking comparison of DTIs; (3) PMF and CMF exhibit inferior performance on all data sets, indicating that the inner product is insufficient to capture the complex relations between drug and target; (4) BRDTI achieves higher AUPR values than CMF, and pNeuRank higher than NeuRank over all data sets, illustrating that adding pair-wise information can boost the performance of the models; (5) on all data sets, RBM has the worst results, indicating that shallow networks without similar information do not make good predictions; (6) NeuRank and pNeuRank capture the nonlinear correlations of latent features via their deep learning strategies; therefore, NeuRank and pNeuRank generally outperform PMF and BRDTI, respectively. Because our models capture the non-liner correlations of the features, they consistently outperform all other baselines. In summary, within the same data set, our methods outperform other competitive approaches, which suggests that the deep learning technique is an effective tool to extract more meaningful features to detect true DTIs.

**Effect of similarity information.** Next, we study how similarity information benefits the prediction of DTIs under settings, $CV_{nd}$. In this experiment, we set a same value for both $\lambda _d$ and $\lambda _t$. The results obtained under the setting, $CV_{nd}$, for new drugs is shown in Table 3. The best results are shown in bold.

**Table 3:** AUC and AUPR values of all methods on five data sets under the setting $CV_{nd}$

Data sets		PMF	CMF	RBM	BRDTI	DeepDTIs	NeuRank	pNeuRank	lNeuRank
DrugBank	AUC	0.7816	0.8427	0.6935	0.8520	0.8278	0.8659	0.8683	**0.8694**
	AUPR	0.5371	0.6188	0.5607	0.6258	0.6043	0.6236	0.6285	**0.6317**
Enzymes	AUC	0.8259	0.8616	0.5093	0.8667	0.8538	0.9229	0.9268	**0.9283**
	AUPR	0.5183	0.5591	0.4380	0.5604	0.5325	0.5775	0.5826	**0.5863**
Ion Channels	AUC	0.8086	0.8640	0.6476	0.8754	0.8539	0.9092	0.9118	**0.9142**
	AUPR	0.5273	0.5601	0.4269	0.5896	0.5634	0.6098	0.6130	**0.6151**
GPCRs	AUC	0.5645	0.6328	0.5006	0.6379	0.5993	0.6557	0.6532	**0.6598**
	AUPR	0.3374	0.3742	0.3059	0.3865	0.3570	0.3988	**0.4020**	0.3781
Nuclear Receptors	AUC	0.6095	0.6537	0.5583	0.6610	0.6328	**0.6645**	0.6573	0.6522
	AUPR	0.2396	0.3517	0.1470	0.3845	0.3329	**0.4882**	0.4796	0.4605

The best results are shown in bold

The results in Table 3 show that our methods, compared with other methods under different settings, yield optimal AUC and AUPR values, indicating that our method, with similarity information, achieves consistently accurate prediction results across all data sets. Compared with the performance in the setting $CV_{dt}$, after including similarity metrics, our models, BPDTI, and CMF achieve comparable results in the setting $CV_{nd}$, indicating that adding similarity information to the models is very effective for finding new DTIs. Therefore, it is clearly seen that considering multiple similarities is critical for optimal prediction performance.

To further illustrate the similarity information effects on the prediction of DTIs, we conducted experiments using the DrugBank data sets. In these experiments, we randomly selected one interaction of each drug as testing data and the remainder as training data. Then, we ranked all unobserved DTIs by our trained models. We compared NeuRank with its simplified version without similarity information and selected three examples. The experimental results are shown in Table 4.

**Table 4:** Example prediction of similarity effect

Drug ID	top	NeuRank without similarity	NeuRank with similarity
DB00122	1	Q05586	**Q8NE62**
	2	P18505	P00918
	3	P10635	Q05586
	4	O60656	Q9Y5N1
DB01151	1	Q8N142	**P10635**
	2	**P10635**	Q9H4B7
	3	P00519	Q8N142
	4	P69905	O60603
DB08271	1	O14764	**P39900**
	2	Q16539	Q02318
	3	P08581	P35916
	4	**P39900**	O14764

True targets are marked in bold

From Table 5, it is seen that, compared with the simplified version without similarity information, the predictions of NeuRank, in all cases, are always more accurate. Without similarity information, not only does the previous method incorrectly predict a target in the top-4 results in the first case, but also achieves worse results in the other cases. In summary, similarity regularization shows strong improvement over our method.

**Fig. 4:**
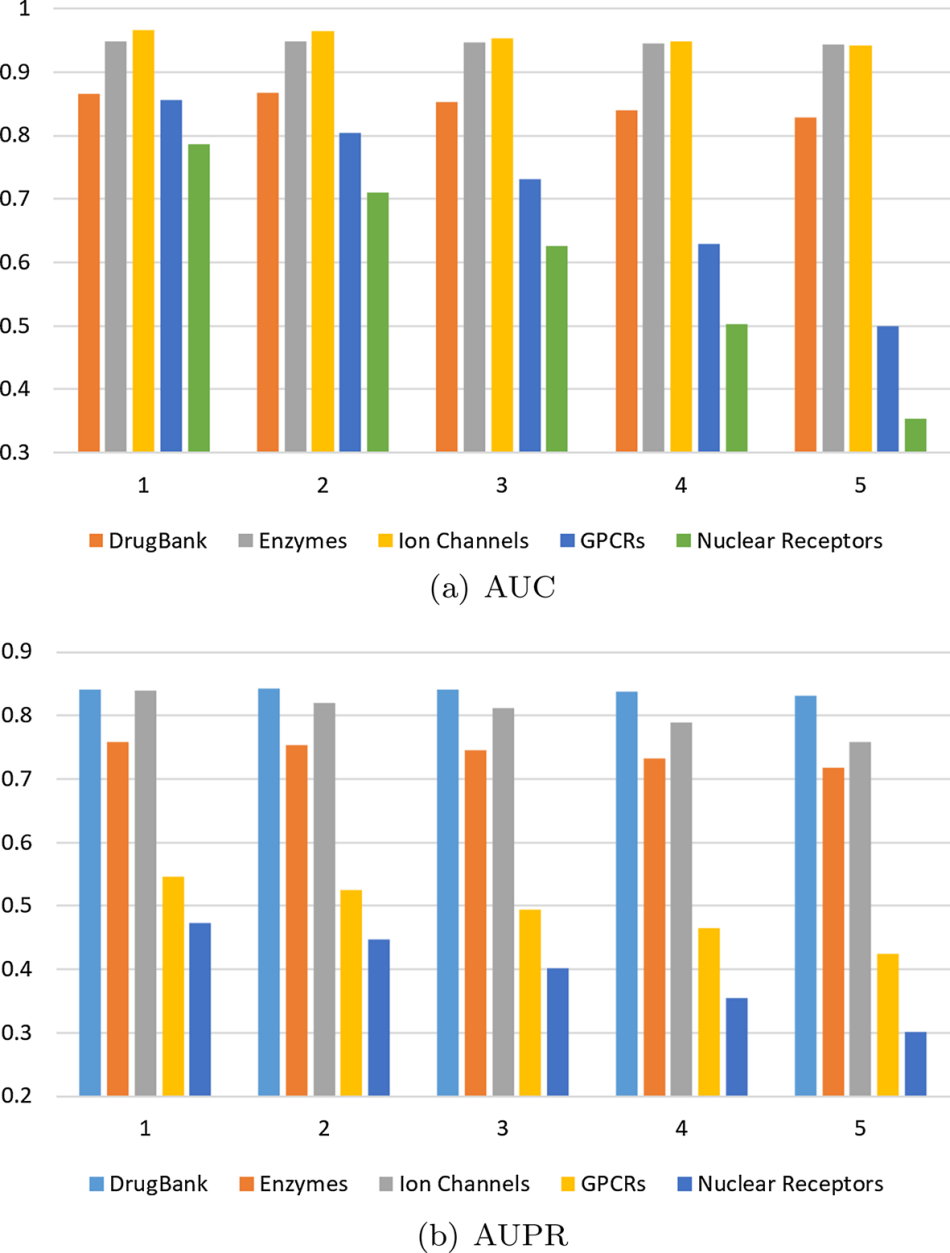
Effect of hidden layer depth of our models on five data sets under the setting $CV_{dt}$. It shows the performance of AUC and AUPR as the number of hidden layers goes from one to five by step one

**Effect of hidden layers depth (***l***).** In addition, we studied the impact of hidden layers depth on the prediction of DTIs for our models. In this experiment, the number of hidden layers goes from one to five by step one under the setting, $CV_{dt}$, on all data sets. Figure 4 shows the performance of AUC and AUPR as the number of depth is changed.

**Table 5:** Effect of embedding size

Data sets	***k***	NeuRank	pNeuRank	lNeuRank
DrugBank	8	0.8737	0.8785	0.8816
	16	0.8911	0.8948	0.8972
	32	0.8941	0.8983	0.9007
	64	0.8858	0.8894	0.8920
	128	0.8415	0.8463	0.8502
Enzymes	8	0.9469	0.9485	0.9502
	16	0.9479	0.9496	0.9519
	32	0.9483	0.9502	0.9539
	64	0.9417	0.9434	0.9456
	128	0.9036	0.9077	0.9118

It shows the performance of AUC as the number of embedding size is changed

As seen in Fig. 4, on the large data sets, DrugBank and Enzymes, the performance of NueRank remains stable as depth increases; on the small data sets, Ion Channels, GPCRs and Nuclear Receptors, the performance of NueRank decreases as depth increases. Deep neural networks have a strong ability to express features; however, for the small data sets, too many parameters can easily lead to over-fitting. Therefore, we conclude that a sensible number of hidden layers is indeed helpful for improving the model.

**Effect of embedding size (***k***).** Finally, we illustrate the effects different embedding sizes (latent feature sizes) have on prediction under the setting $CV_{dt}$ in our proposed models. For simplicity, we conducted experiments on two largest data sets: DrugBank and Enzymes, and use AUC to evaluate. In this experiment, the embedding size was selected within the range $\{8, 16, 32, 64, 128\}$. The effect embedding size has on the performance of our models is shown in Table 4.

As seen from Table 4, our methods achieve best results when $k=32$. And *k* increases, there is a clear increasing trend in the AUC values until the maximum is reached at $k=32$; then, at $k=64$, there is a slight decrease. Thus, it is seen that an embedding size that is too large causes the model to be over-fitting; an embedding size that is too small causes the model to be under-fitting. Consequently, an appropriate size is important for the model to learn meaningful and accurate features and perform well.

## Conclusion

Prediction of DTIs plays an import role in the drug discovery process. We proposed three novel methods, NeuRank, pNeuRank, and lNeuRank, to predict the interaction probability. Our models are neural network architectures, which have a powerful ability to effectively learn nonlinear and deep features for predicting DTIs. In addition, especially for new drugs and targets, some similarity information is added to our models for better performance. Experimental results show that, compared with baseline approaches, our methods achieve better performance and higher quality. What is more, our methods can provide useful hits for further biological study of drug discovery and development.

In future work, first, we plan to integrate more biological information to further improve our models; second, because similarity computation plays a critical role in learning accurate latent features, we plan to explore other nonlinear techniques to combine similarity matrices for drugs and targets; finally, for wider application, we will try to incorporate our models with other deep learning models.

## Data Availability

DrugBank Database is available at: http://www.drugbank.ca. Nuclear Receptors, G-Protein-Coupled Receptors (GPCRs), Ion Channels and Enzymes data sets, are available at: http://web.kuicr.kyoto-u.ac.jp/supp/yoshi/drugtarget/.

## Abbreviations

DTIs: Drug–target interactions
AUC: Area Under the receiver operator characteristic curve
AUPR: Area under the precision-recall curve

## Footnotes

1 http://www.drugbank.ca.
